# The hGFAP-driven conditional TSPO knockout is protective in a mouse model of multiple sclerosis

**DOI:** 10.1038/srep22556

**Published:** 2016-03-01

**Authors:** Daniel J. Daugherty, Olga Chechneva, Florian Mayrhofer, Wenbin Deng

**Affiliations:** 1 Department of Biochemistry and Molecular Medicine, School of Medicine, University of California, Davis, CA, USA; 2 Shriners Institute for Pediatric Regenerative Medicine, Sacramento, CA, USA; 3 Medical College, Hubei University of Arts and Science, Xiangyang, Hubei, China

## Abstract

The mitochondrial translocator protein (TSPO) has been implicated in CNS diseases. Here, we sought to determine the specific role of TSPO in experimental autoimmune encephalomyelitis (EAE), the most studied animal model of multiple sclerosis (MS). To fundamentally elucidate the functions of TSPO, we first developed a viable TSPO knockout mouse. A conditional TSPO knockout mouse was generated by utilizing the Cre-Lox system. We generated a TSPO floxed mouse, and then crossed this mouse with a Cre recombinase expressing mouse driven by the human glial fibrillary acidic protein (hGFAP) promoter. The resultant mouse was a neural linage line specific TSPO knockout. The loss of TSPO in the CNS did not result in overt developmental defects or phenotypes. The TSPO−/− mouse showed a decrease in GFAP expression, correlating with a decrease in astrogliosis in response to neural injury during EAE. This decrease in astrogliosis was also witnessed in the lessening of severity of EAE clinical scoring, indicating an *in vivo* functional role for TSPO in suppressing EAE. The TSPO−/− mouse could be a useful tool in better understanding the role of TSPO in CNS disease, and our results implicate TSPO as a potential therapeutic target in MS.

Multiple sclerosis (MS) is a neurodegenerative disorder which affects 2.1 million people worldwide. It is marked by an autoimmune response against neural tissue by infiltrating peripheral immune cells. There is no known cure for MS; however there are multiple forms of therapy which aid in curbing of MS symptoms. These therapies target the immune response or the initial cause of the autoimmune response, and do not directly aid in central nervous system (CNS) repair. We have previously shown that the translocator protein (TSPO) plays a role in MS symptom and CNS repair. TSPO has been shown to be upregulated in various forms of CNS disorders including MS, Alzheimer’s disease, Huntington’s disease, stroke, traumatic brain injury, and Parkinson’s disease^1^,^2^,^3^,^4^,^5^. TSPO has also been shown to be upregulated in activated cells, including immune cells, microglia, and astrocytes^1^,^6^. The role TSPO plays in the CNS is not well understood. Located on the outer mitochondrial membrane, TSPO has been shown to play a role in cell activation, opening of the mitochondrial permeability transition pore, and steroidogenesis^7^,^8^. How these processes factor into CNS injury or disease could play an important role in better understanding the CNS and in drug development. To elucidate the functions of TSPO, we developed the first CNS specific TSPO knock-out mouse. A conditional TSPO knockout mouse was generated by utilizing the Cre-Lox system.

We first generated a TSPO floxed mouse, and crossed this mouse with a Cre recombinase expressing mouse driven by the human Glial Fibrillary Acidic Protein (hGFAP) promoter. The resultant mouse was a neuroectoderm lineage specific TSPO knockout mouse, with active Cre expression in astrocytes and neural precursors^9^. We found no developmental issue caused by the loss of neural TSPO activity. However, we discovered a novel modulation of astrocyte function that contributes to a decrease in symptoms in the mouse model of MS, experimental autoimmune encephalomyelitis (EAE). Thus, we first developed a viable knockout mouse of mitochondrial TSPO and showed that TSPO deletion is protective in a mouse model of MS, indicating that mitochondrial TSPO is a novel therapeutic target for MS. More broadly, the TSPO−/− mouse could be a useful tool in better understanding both the fundamental role of TSPO in the CNS and its function in CNS injury or disease.

## Methods

### Generation of TSPO^Fl^°^x/Fl^°^x^ animal

Experiments were carried out in accordance with the National Institutes of Health guidelines for the use of laboratory animals. All animal protocols were approved by the University of California-Davis Institutional Animal Care and Use Committee. All efforts were made to minimize the numbers of animals used and to ensure minimal suffering. A vector containing flox sites flanking Exons 2 and 3 of the *Tspo* gene was created. This vector was electroporated into C57BL/6 ES cells. Cells were screened through PCR. Cells positive for homologous recombination are injected into blastocytes and transferred for embryo growth. Chimera animals are measured for germline transmission. Animals experiencing germline transmission are crossed with each other to generate heterozygous mice. TSPOflox heterozygous mice were crossed to generated homozygous mice. Homozygous TSPOflox mice were crossed with GFAPcre mice to generate TSPO^Flox/Flox^/GFAPcre mice.

### Immunohistochemistry

Mice were anesthetized and tissue fixed by transcardial perfusion. The lumbar section of the spinal cord was isolated and placed in 4% PFA overnight. The tissue was then cryoprotected in 30% sucrose and frozen in OCT (Sakura Finetek, Torrance, CA). The tissue was cut into 20 μm sections and kept in PBS at 4 °C until stained.

For immunostaining, tissue sections were post-fixed in 4% PFA for 30 min and washed with PBS. Non-specific binding was blocked using 5% goat serum, and the tissue was perforated with 0.5% triton-X in PBS, before probing with antibodies for TSPO (Epitomics), GFAP (Sigma-Aldrich or DAKO), Cre recombinase (Millipore) or FITC conjugated lectin from Lens culinaris (Sigma). The tissue was then washed in PBS and stained with a secondary antibody conjugated to Alexa Fluor 488 or Alexa Fluor 555 for 2 hrs. Nuclear staining was done with DAPI Fluoromount G (SouthernBiotech). Images were taken on a Nikon Eclipse TE 2000-E microscope using a D-Eclipse C1si camera (Nikon Instruments Inc., Melville, NY).

### Induction of EAE

EAE was induced in 10-week-old female mice by injecting an emulsion of 300 μg of MOG peptide in complete Freund’s adjuvant (CFA) subcutaneously on either hind flank as two injections. In addition, 250 ng of pertussis toxin was injected intraperitoneally on the same day as MOG-CFA, and another dose was administered after 48 hrs. Body weights of mice were recorded before MOG-CFA injection and then continuously at 2-day intervals. Disease development was monitored daily, and the severity of clinical signs was scored based on a standard neurological scoring system for EAE as follows: 1, limp tail or waddling gait; 2, limp tail and ataxia; 2.5, single limb paresis and ataxia; 3, double limb paresis; 3.5, single limb paralysis and paresis of second limb; 4, full paralysis of two limbs; 4.5, moribund; and 5, dead. Scoring was performed in a blinded fashion.

### mRNA isolation and analysis

Mice were anesthetized with ketamine:xylazine (100:10 mg/kg) and perfused through the heart with a phosphate-buffered saline (PBS). Lumbar spinal cords were carefully excised and stored separately in liquid nitrogen. Total RNA was isolated from spinal cord tissue using RNeasy Lipid Tissue Mini Kit (Qiagen) following the standard protocol. For quality control, RNA purity was verified using the OD260/280 ratio to be between 1.8 and 2.0. Total RNA (1 μg) was reverse-transcribed to cDNA using Multiscribe^TM^ reverse transcriptase (Applied Biosystems). qPCR for GAPDH (Mm99999916_s1), TSPO (Mm00437828_m1), TNF-α (Mm00443258_m1) and CXCL10 (Mm00445235_m1) was performed in duplicates using the TaqMan gene expression assay (Applied Biosystems) using a Roche Lightcycler 480. All samples were analyzed and normalized with the expression level of GAPDH, and quantification of fold-change was performed utilizing the 2^−ΔΔ^Ct method.

### Western Blotting

GFAP protein expression was examined by Western Blot as described previously^10^. Briefly, mice were anesthetized and perfused through the heart with PBS. Lumbar spinal cords were isolated and tissue was homogenized in RIPA buffer contained protease inhibitors (Roche). 20 μg of protein was denatured in SDS buffer containing 50 mM DTT and heated for 5 min at 90 °C. Proteins were separated on 11% Tris-HCL gels and transferred to nitrocellulose membranes (BioRad). Membranes were blocked using 5% milk in PBS with 0.1% Tween-20 for 1 h at RT and incubated overnight at 4 °C with primary antibodies: rabbit anti-GFAP (1:1000, Boster Biological Thechnology) and mouse anti-β-actin (1:2000, Abcam). Membranes were washed and incubated with secondary antibodies: IRDye 800CW goat anti-mouse and IRDye 680RD goat anti-rabbit (Li-Cor) for 1 h at RT. After washing, stained proteins were visualized using Odyssey Infrared Imager (Li-Cor). ImageJ (NIH) was used to quantify band density. Protein abundance was normalized to β-actin and expressed as amount of protein relative to control.

### Data analysis

All data represent the mean ± SEM. Each experimental group had at least 8 mice. Statistical differences were assessed by analysis of variance (ANOVA) with Tukey *post hoc* analysis for multiple comparisons. Student’s *t* test was used when only two independent groups were compared. For data not satisfying assumptions of normality and homogeneity of variance, a nonparametric Mann–Whitney test was used. P values of less than 0.05 were considered significant.

## Results

### Generation of TSPO^Flox/Flox^ animal

**Figure 1:**
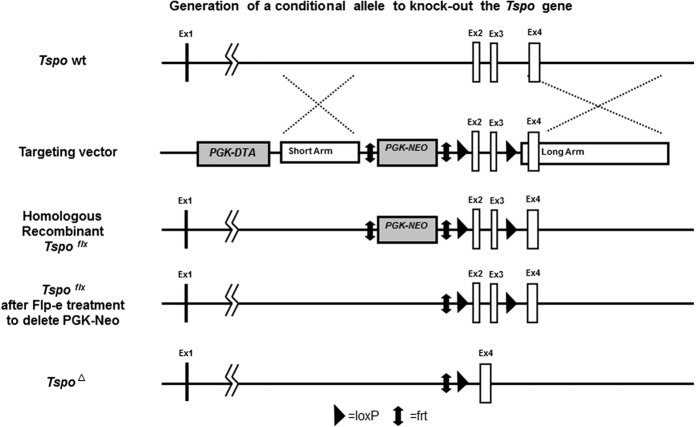
Generation of a conditional allele, via a sequence replacement strategy to knock-out the *Tspo* gene. The construct contains loxP sites that flank exons 2 & 3, a 2.8 kb 5′ short arm of homology, a 7.3 kb 3′ long arm of homology, a Diphtheria Toxin A (DTA) cassette, and a Neomycin (Neo) cassette flanked by frt sites for selective deletion. The Neo element allows for positive selection in ES cells, while the DTA element permits negative selection in ES cells. After homologous recombination of our conditional knock-out construct, the PGK-Neo can be excised via Flp-e electroporation. The *Tspo* gene has normal expression until Cre-mediated deletion of exons 2 & 3. This recombination creates a frameshift mutation and a premature stop, rendering the *Tspo* gene inactive.

The TSPO^Flox/Flox^ mice were generated through homologous recombination. As shown in Fig. 1, a TSPO targeting vector was generated containing LoxP sites flanking exons 2 and 3. The vector was inserted into mouse germline cells, and recombination was measured through neomycin resistance. Mice containing the LoxP sites were breed with each other to generate a homologous TSPO^Flox/Flox^ animal. Genetic composition was checked through PCR and gel electrophoresis.

### Generation of conditional TSPO^−/−^ animal

**Figure 2:**
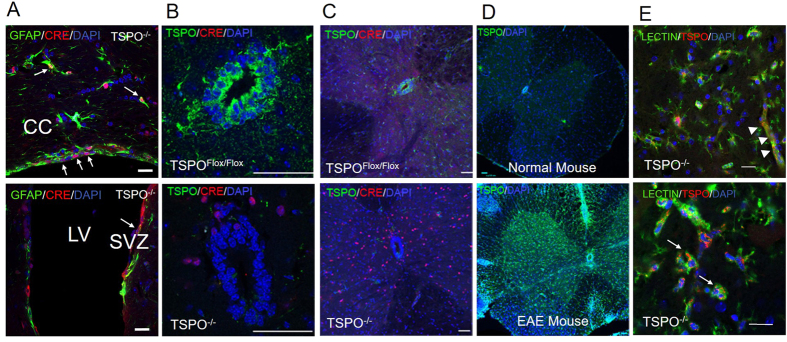
Cre and TSPO expression in the CNS in TSPO^−/−^ mice. Cre expression was observed in co-localization with GFAP + astrocytes and neural precursors in the brain (**A**) and spinal cord (**B**) in TSPO^−/−^ mice. TSPO^Flox/Flox^ mice showed normal expression of TSPO (**C**), and the expression was strongest in the central canal (**B**). TSPO^−/−^ mice showed a marked decrease in TSPO expression (**C**), particularly evident around the central canal (**B**). Cre expression was seen in TSPO^−/−^ mice, but not in TSPO^Flox/Flox^ mice (**B**,**C**). TSPO expression was generally low in the normal mouse CNS (**D**). There was a marked increase in TSPO expression in response to EAE (**D**). TSPO expression was seen in cells of the non-neural linage, microglia (arrows) and endothelial cells (arrowheads), in the hGFAP-driven conditional TSPO^−/−^ mice (**E**). CC, corpus callosum; LV, lateral ventricle; SVZ, subventricular zone. Scale bars: 20 μm for E; 50 μm for A and B; 100 μm for C and D.

The conditional TSPO^−/−^ animal was generated through cross breeding the TSPO^Flox/Flox^ mice with mice which contained the FVB-Tg(GFAP-cre)25Mes/J gene. The FVB-Tg(GFAP-cre)25Mes/J gene controls the expression of Cre recombinase by the human GFAP promoter. Animals containing Cre and homologous for TSPO^Flox/Flox^ were considered knockout mice, and were selected through PCR. As described previously, mice containing the hGFAP promoter express Cre in cells from the neural lineage^9^. Cre expression was seen in GFAP expressing astrocytes and neural precursors in the subventricular zone (Fig. 2A, arrow) and cells lining the central canal of the spinal cord (Fig. 2B). Cre+cells were detected in the spinal cord of TSPO^−/−^ mice (Fig. 2C). TSPO^−/−^ mice did not demonstrate any developmental defects or apparent phenotypes. The lack of developmental abnormalities was evident in all of the generated TSPO knockout (KO) mice maturing to adulthood, with no apparent phenotype. Immunohistochemistry showed no apparent differences in the developing and adult knockout mice compared to wild-type (WT) animals.

### TSPO expression in the CNS

As shown in Fig. 2D, minimal TSPO expression is seen in normal mouse, limited to the ependymal cells and microglia. Upon initiation of EAE, TSPO expression is drastically increased, particularly in microglia and astrocytes. TSPO expression in the central canal was lost in the TSPO^−/−^ mice, and the only cells which demonstrated TSPO expression were microglia and microvascular endothelial cells. (Fig. 2E). Cre expression verified that the hGFAP Cre promoter was indeed initiating Cre transcription in the neural lineage and knocking out TSPO.

### EAE in TSPO^−/−^ mice

**Figure 3:**
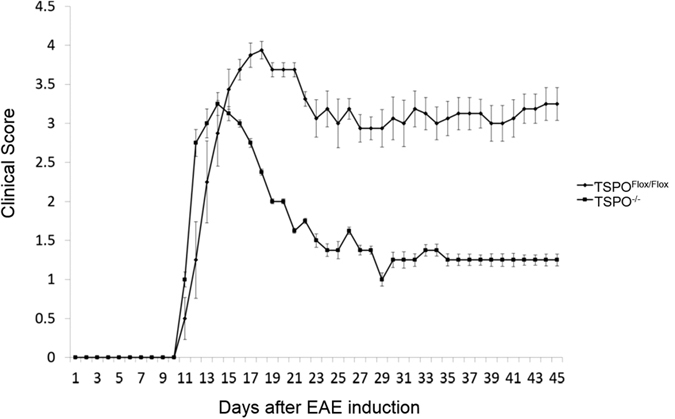
Clinical scores of TSPO^Flox/Flox^ and TSPO^−/−^ mice during EAE. Clinical scores during EAE were decreased in the TSPO^−/−^ group compared to the TSPO^Flox/Flox^ group. There was a significant difference in the groups starting at day 16 and continuing throughout the experiment (n = 8 animals per group).

As shown in Fig. 3, EAE initiated in TSPO^Flox/Flox^ mice and TSPO^−/−^ mice displayed a significant difference between the two groups. TSPO knockout mice showed a decrease in the severity of clinical scoring, with a decrease in maximal scores and time of paralysis. The KO group also experienced increased survivability.

### GFAP expression in TSPO^−/−^

**Figure 4:**
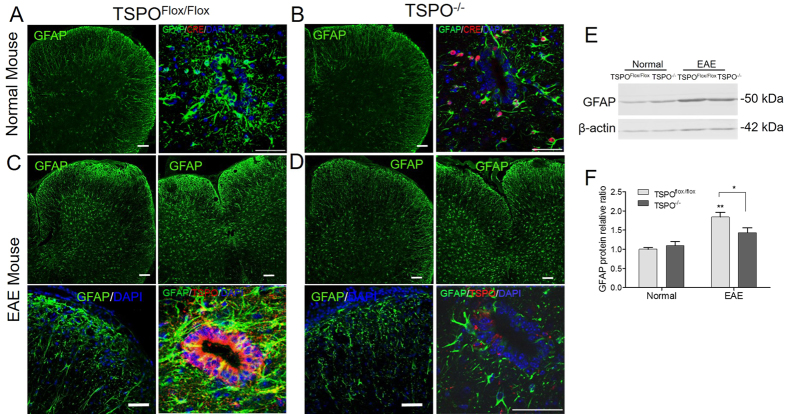
GFAP expression in the CNS in normal mice and during EAE. GFAP expression in normal TSPO^Flox/Flox^ (**A**) and TSPO^−/−^ (**B**) mice was seen throughout the CNS. GFAP expression was contained to the grey and white matter astrocytes and the cells of the central canal. There was no Cre expression seen in TSPO^Flox/Flox^ (**A**) while Cre + cells were present in TSPO^−/−^ mice (**B**). GFAP expression was increased in EAE mice (**C**). TSPO^Flox/Flox^ mice showed an increase in GFAP and TSPO expression, in particular within the cells of the central canal. Central canal cells also co-localized with TSPO, along with astrocytes within the grey matter of the spinal cord. TSPO^−/−^ mice retained lower GFAP expression upon induction of EAE, and TSPO expression was not seen as increased in GFAP expressing cells (**D**). Scale bars: 100 and 50 μm for (**A,B**) 100, 100, 50 and 50 μm for (**C**,**D**). Representative Western Blot shows increase in GFAP protein level in EAE mice with a reduction seen in TSPO^−/−^ group (**E**). Quantitative analysis of GFAP protein shows dramatic increase in GFAP expression in TSPO^Flox/Flox^ mice after EAE. Reduced GFAP expression is detected in TSPO^−/−^ mice (**F**). n = 7; *p < 0.05; **p < 0.01.

GFAP protein analysis showed no dramatic differences in GFAP expression and distribution in the spinal cord tissues TSPO^Flox/Flox^ vs TSPO^−/−^ mice (Fig. 4A,B,E,F). Although less intense GFAP expression was observed around central canal of the spinal cord where it was co-localized with Cre expression in TSPO^−/−^ mice (Fig. 4A,B). The differences in GFAP expression was made evident in EAE mice (Fig. 4C,D). While a massive astrogliosis was observed in the ventral spinal cord of TSPO^Flox/Flox^ mice, less GFAP immunoreactivity was detected in KO animals. Protein analysis showed two-fold increase in GFAP protein level in TSPO^Flox/Flox^ mice after EAE, no significant differences were detected TSPO^−/−^ mice between normal and EAE groups (Fig. 4E,F). TSPO^−/−^ mice demonstrated decreased GFAP expression, particularly around the central canal of the spinal cord, compared to TSPO^Flox/Flox^ mice. Thus, TSPO KO did not significantly alter the level of GFAP protein in non-immunized mice, but TSPO^−/−^ mice showed reduced astrogliosis and clinical symptoms in EAE.

### mRNA expression during EAE

**Figure 5:**
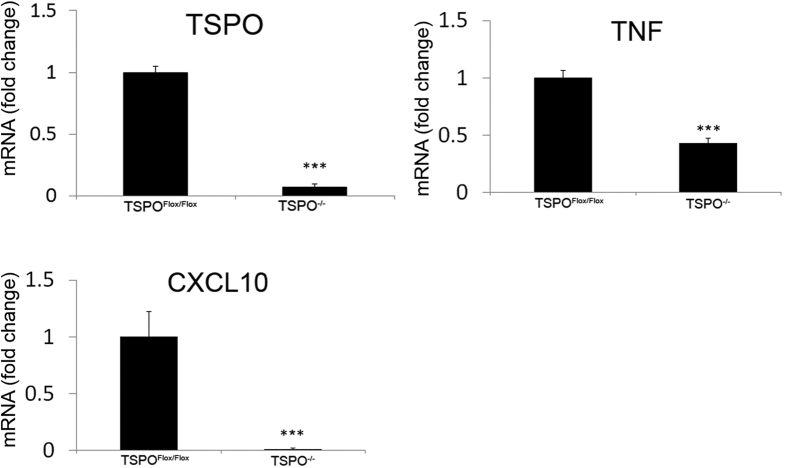
mRNA expression during EAE of TSPO^Flox/Flox^ and TSPO^−/−^ mice. There was a significant decrease in TSPO mRNA in the TSPO^−/−^ mice compared to the TSPO^Flox/Flox^ group. Significant decreases in mRNA levels of cytokines TNFα and CXCL10 were also seen. ***p < 0.001.

As shown in Fig. 5, mRNA of TSPO was decreased 93% in the TSPO^−/−^ group compared to the TSPO^Flox/Flox^ group. The pro-inflammatory cytokine TNFα also demonstrated a 50% decrease in the TSPO^−/−^ group compared to control. The anti-inflammatory effect was also seen in the decrease in the 99% decrease of the cytokine CXCL10.

## Discussion

The role of TSPO in the CNS is not well understood. Primarily thought of as participating in steroidogenesis, its increased expression during CNS disease also suggests that it plays a role in response to injury. Previous studies have shown that pharmaceutical manipulation of TPSO could lead to an increase in steroidogenesis within the CNS, as well as a protective and anti-inflammatory effect, both localized within glia cells and peripheral immune cells^11^. A recent study has shown that deletion of TSPO in Leydig cells results in no change of steroid production^12^. Additionally, an adrenal specific KO did not affect mPTP function^13^. A constitutive TSPO KO was not only entirely viable, it also displayed no phenotype^14^. The recent understanding of TSPO function has required a new paradigm for the role of TSPO in the CNS. To better understand the role of TPSO in the CNS, a CNS specific TSPO^−/−^ was generated. The TSPO^Flox/Flox^ mouse was generated through the insertion of LoxP sites before exon 2 and after exon 3, resulting in a frame shift mutation and a premature stop codon. LoxP sites were inserted through homologous recombination with a cassette injected in germline cells. Recombinated cells were selected through neomycin resistance and injected for gestation. Mice with TSPO^Flox/Flox^ genes were selected through the use of PCR verification. Heterozygous TSPO^Flox/Flox^ mice were breed with each other to generate homozygous TPSO ^Flox/Flox^ animals. Homozygous TSPO ^Flox/Flox^ mice were then breed with mice containing the hGFAP protein driven Cre recombinase. The hGFAP promoter is active in cells of the neural lineage during development. This leads to Cre expression and recombination of LoxP sites in all cells of the neural lineage, resulting in a CNS specific TSPO KO^9^. In the adult mouse, Cre expression was mostly seen in astrocytes and ependymal cells. TSPO expression in the normal adult mouse is generally low, with increased expression in ependymal cells which constitute the central canal of the spinal cord. TSPO^Flox/Flox^/hGFAPcre mice showed highly down regulated TPSO expression, with minimal expression of TPSO in the central canal. Cre expression was seen throughout the CNS with the majority of expression seen in GFAP^+^ astrocytes, and ependymal cells. This demonstrates that Cre expression is being driven by the GFAP promoter, and resulting in excision of the floxed DNA and effective deletion of the TSPO gene.

We generated the conditional GFAP-specific TSPO knockout mouse in order to study the role of astroglial TSPO signaling in EAE *in vivo* under the inflammatory condition in the CNS. However, all cells derived from the neuroectoderm lineage underwent a GFAP-expression stage and should have also been conditionally knocked out of TSPO during early neurogenesis^9^. Moreover, adult neurogenesis has been suggested to be mainly derived from GFAP expressing progenitor cells. Therefore, in our knockout mice, all cells from the neuroectoderm lineage are TSPO^−/−^, while the only actively Cre expressing cells are GFAP+astrocytes and neural precursor cells.

Previous studies have demonstrated an increase in TSPO expression in response to injury^6^,^15^,^16^. We have previously shown an increase in TSPO expression in the mouse model of MS, EAE^16^. Upon induction of EAE, TSPO expression is elevated in astrocytes; however, no TSPO expression in astrocytes was seen in the knockout animals. TSPO is also increased in the central canal, which was negated in the knockout. This once again proves the effectiveness of the TSPO-KO, even with induced expression.

The role TSPO plays in MS is not completely understood. It is known that there is an increase in TSPO expression in astrocytes and microglia in individuals suffering from MS. The use of TSPO ligands has shown to decrease microglia activation, and inflammatory cytokine production^17^. TSPO ligands have also been demonstrated to increase the production of neurosteroids by astrocytes^8^. The loss of TSPO in astrocytes proved to show a decrease in astrogliosis. Although no differences in GFAP expression were seen in healthy TSPO^−/−^ mice compared to WT, WT mice demonstrated a vast increase in GFAP expression in response to EAE, while this increase was lessened in the TSPO^−/−^ cohorts. This decrease in GFAP expression corresponds to a decrease in astrogliosis, suggesting that TSPO plays a role in astrocyte activation. The decrease in GFAP expression was seen throughout the spinal cord. The central canal also experienced a decrease in GFAP, which is normally a location of strong astrogliosis in response to EAE induction.

The function astrocytes play in response to neural injury is not well understood, but it is known that an overabundance of astrogliosis can lead to glia scarring and permanent damage. The decrease in astrogliosis demonstrated in the TSPO^−/−^ mice could lead to a protective mechanism. The clinical scores of the TSPO^−/−^ mice showed a decrease in severity of EAE. This suggests that the loss of TSPO in the CNS leads to a decrease in inflammatory damage. It is possible that TSPO is necessary for astrogliosis, and the production of inflammatory cytokines. The loss of TSPO causes a decrease in harmful astrocyte function during EAE.

The decrease in astrocyte activation was corroborated with analysis of mRNA steady-state levels. The amount of TSPO mRNA was decreased in the TSPO^−/−^ group correlating with the loss of the *Tspo* gene in the neural lineage. The decrease in TSPO mRNA was associated with a decrease in TNFα mRNA as well as a decrease in CXCL10. Previous studies have shown that TNFα is correlated with astrocyte activation as well as TSPO modulation can affect TNFα production^18^,^19^. Astrocyte production of CXCL10 has also been shown to attract peripheral immune cells during EAE, suggesting that a decrease in astrocyte activation may cause a decrease in CNS infiltration^20^.

It has been much debated on the importance of TSPO for steroid formation and production. Previous studies have implicated TSPO in the induction and maintenance of steroidogenesis^1^,^2^,^3^,^4^,^5^. Further studies evaluated the function of TSPO in the physiology of steroid formation and production but also at the pathophysiology of a number of human diseases^1^,^2^,^3^,^4^,^5^. Recent studies have shown that deletion of TSPO does not lead to embryonic lethality and does not alter steroid metabolism, suggesting that TSPO is not critically involved in the regulation steroid synthesis and function^10^. In particular, Banati *et al.* has just reported that mice with a global TSPO knockout are viable with normal growth, lifespan, cholesterol transport, blood pregnenolone concentration, protoporphyrin IX metabolism, fertility and behavior^21^. However, changes in TSPO levels have been previously linked to changes in brain steroid levels and associated with neuropathology, including neurodegenerative disorders, brain injury, anxiety, and mood disorders. TSPO drug ligands are under investigation in the clinic for treatment of psychiatric and neurologic disorders.

CNS injury or inflammation results in the induction or enhanced expression of TSPO in glial cells. Several TSPO ligands have been reported to decrease reactive astrogliosis after CNS injury or disease. However, since these TSPO ligands are neuroprotective, their effects on reactive astrogliosis may be an indirect or secondary result due to reduced neural damage. It would be interesting to assess whether TSPO ligands could directly regulate reactive astrogliosis in absence of neuronal loss or injury. Furthermore, TSPO is also involved in controlling neural inflammation and microglial activation. Therefore, TSPO may indeed represent a target to reduce astrogliosis, but there may well be compensatory mechanisms. In addition, TSPO may also be necessary to maintain glial function in the CNS. Nonetheless, GFAP is a robust marker for astrogliosis, and the decrease in GFAP expression indicates a decrease in astrogliosis in response to the deletion of TSPO. Future studies are warranted to further address the importance of the GFAP decrease and the consequence of GFAP decrease in gray matter as well as white matter in the TSPO^−/−^ mice.

In conclusion, we report that in an experimental model of MS, a human GFAP driven conditional TSPO KO shows a decrease in the severity of the clinical scoring and in the mRNA levels of the cytokines TNFα and CXCL10 in the spinal cord. These findings suggest that TSPO KO in astrocytes decreases neuroinflammation and is neuroprotective. The generation of the first CNS specific TSPO knockout animal was accomplished through the utilization of the Cre/Lox system. The loss of TSPO in the CNS did not result in overt developmental defects or phenotypes. The TSPO^−/−^ animal demonstrated a decrease in GFAP expression, correlating with a decrease in astrogliosis in response to neural injury during EAE. This decrease in astrogliosis was also witnessed in the lessening of severity of EAE clinical scoring, indicating a role for TSPO in suppressing EAE. The data suggest that the role of TSPO in neurosteroid production may not be as important as once thought. Our findings suggest that TSPO may be required for inflammatory cytokine production in response to injury in EAE mice. The TSPO^−/−^ mouse could be a useful tool in better understanding the role of TSPO in CNS injury or disease.

## Additional Information

**How to cite this article**: Daugherty, D. J. *et al.* The hGFAP-driven conditional TSPO knockout is protective in a mouse model of multiple sclerosis. *Sci. Rep.* **6**, 22556; doi: 10.1038/srep22556 (2016).
